# Interactive effects of global climate change and pollution on marine microbes: the way ahead

**DOI:** 10.1002/ece3.565

**Published:** 2013-04-23

**Authors:** Francisco J R C Coelho, Ana L Santos, Joana Coimbra, Adelaide Almeida, Ângela Cunha, Daniel F R Cleary, Ricardo Calado, Newton C M Gomes

**Affiliations:** 1 Department of Biology and CESAM, University of Aveiro, Campus de Santiago3810-193, Aveiro, Portugal; 2 Central Laboratory of Analysis, University of Aveiro, Campus de Santiago3810-193, Aveiro, Portugal

**Keywords:** Climate change, interactive effects, pollution, microbial communities, molecular biology

## Abstract

Global climate change has the potential to seriously and adversely affect marine ecosystem functioning. Numerous experimental and modeling studies have demonstrated how predicted ocean acidification and increased ultraviolet radiation (UVR) can affect marine microbes. However, researchers have largely ignored interactions between ocean acidification, increased UVR and anthropogenic pollutants in marine environments. Such interactions can alter chemical speciation and the bioavailability of several organic and inorganic pollutants with potentially deleterious effects, such as modifying microbial-mediated detoxification processes. Microbes mediate major biogeochemical cycles, providing fundamental ecosystems services such as environmental detoxification and recovery. It is, therefore, important that we understand how predicted changes to oceanic pH, UVR, and temperature will affect microbial pollutant detoxification processes in marine ecosystems. The intrinsic characteristics of microbes, such as their short generation time, small size, and functional role in biogeochemical cycles combined with recent advances in molecular techniques (e.g., metagenomics and metatranscriptomics) make microbes excellent models to evaluate the consequences of various climate change scenarios on detoxification processes in marine ecosystems. In this review, we highlight the importance of microbial microcosm experiments, coupled with high-resolution molecular biology techniques, to provide a critical experimental framework to start understanding how climate change, anthropogenic pollution, and microbiological interactions may affect marine ecosystems in the future.

## Introduction

Anthropogenic emissions of carbon dioxide (CO_2_) have increased from approximately 280 ppm (parts per million) in preindustrial times (Indermühle et al. 1999) to nearly 394 ppm in 2012 (NOAA Earth System Research Laboratory, 2012). Levels of CO_2_ in the atmosphere now exceed limits considered natural for most animals and plants (Ehleringer et al. 2002). The best known postulated consequence of an increasing atmospheric CO_2_ concentration is global warming, which may, among other things, lead to sea level changes, promote ocean stratification, and alter the sea-ice extent and patterns of ocean circulation (Doney et al. 2012). In addition to the above, increased atmospheric CO_2_ will also lead to a net air-to-sea flux of CO_2_, thereby reducing seawater pH and modifying the chemical balance among inorganic carbon species. This process, known as ocean acidification, is often referred to as “the other CO_2_ problem” (Henderson 2006). In contrast to other climate change scenarios, ocean acidification is a direct consequence of increased atmospheric CO_2_ and does not depend on uncertainties related to other climate change predictions (Doney et al. 2009).

Although international treaties have been effective in reducing atmospheric concentrations of ozone-depleting substances, increased greenhouse gas concentrations have the potential to affect the spatial distribution of ozone and its exchange between the stratosphere and the troposphere; this, in turn, will influence ultraviolet radiation (UVR) levels reaching the Earth's surface (UNEP 2010, 2012). Higher UVR levels have also been shown to disrupt aquatic food webs and reduce the biological sinking capacity of aquatic environments for atmospheric CO_2_ (Hader et al. 2007; Fabry et al. 2008).

**Figure 1:**
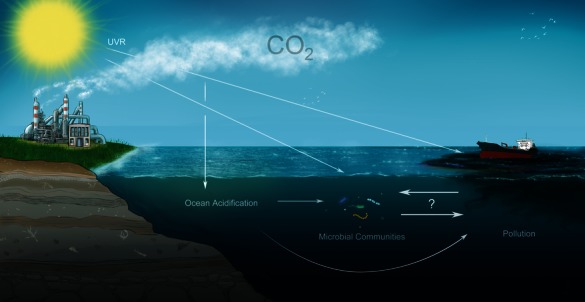
Interactions between ultraviolet radiation, ocean acidification, anthropogenic pollution, and microbial communities. Climate change has the potential to influence pollutant toxicity by acting directly on pollutant chemistry or indirectly by affecting microbial-mediated detoxification.

In addition to the effects of anthropogenic activities on global climate change, fossil fuel combustion, fertilizer use, and industrial activity have adversely affected coastal and open-ocean environments for decades, providing a continuous influx of pollutants [including oil hydrocarbons (OH), pesticides, and heavy metals] into these ecosystems (Doney 2010). With respect to OH, natural seepage alone introduces about 6 × 10^5^ metric tons year^−1^ of crude oil to oceans, representing ∼47% of crude oil entering the marine environment. The remaining 53% results from anthropogenic activities (accidental oil spills, transport activities, refining, storage, and others) (Kvenvolden and Cooper 2003). There is a growing realization among scientists that ocean acidification and increased UVR have the potential to alter contaminant transfer in aquatic food webs, and modify aquatic trophic structures and the biomagnification of contaminants thereby leading to increased toxicity in marine ecosystems (Pelletier et al. 2006; Fabry et al. 2008). Microbial communities play a central role in the global recycling of pollutants. For example, the oil-catabolic versatility of microbes, particularly bacteria, ensures that oceans are not completely covered with an oil film (Head et al. 2006). Despite the importance of microbes in the process of global recycling of anthropogenic pollutants, the potential interactions of ocean acidification, UVR, anthropogenic pollutants, and marine microbial communities have been largely ignored. Little is known about how ocean acidification and increased UVR can interact with anthropogenic pollutants to affect microbial communities and biogeochemical cycling. Moreover, although ocean acidification and increased UVR have the potential to affect microbial assemblages (Riebesell et al. 2007; Liu et al. 2010; Santos et al. 2012), very little is known about the effects on microbial-mediated pollutant detoxification and how this will impact pollutant pathways (Fig. 1). The aim of this review is to present the recent advances in our understanding of the consequences of interactions between ocean acidification, increased UVR, anthropogenic pollutants, and marine microbial communities. We also discuss recent technological advances in molecular microbiology as a means to improving our ability to study potential interactive effects.

## Ocean Acidification and UVR Interactions With Marine Microbial Communities – What We Know So Far

If CO_2_ emissions continue unabated, oceanic pH will decline 0.3 to 0.4 units by the end of this century, and up to 0.7 units in 2300 (Caldeira and Wickett 2003). When CO_2_ dissolves in seawater, carbonic acid (H_2_CO_3_) is formed and quickly dissociates into hydrogen (H^+^) and bicarbonate (HCO_3_^−^) ions. A hydrogen ion can then react with a carbonate ion (CO_2_^−3^) to form bicarbonate. This process leads to increased partial pressure (*p*CO_2_), increased concentrations of H_2_CO_3_, HCO^−^_3_, and H^+^, and reduced concentrations of CO_3_^−2^ (Fabry et al. 2008). These changes in carbonate chemistry have serious implications for marine organisms that depend on minerals such as calcite and aragonite to produce shells and skeletons (e.g., corals, mollusks, echinoderms, and crustaceans). Indeed, the available data suggests that calcification rates will be affected under future *p*CO_2_ scenarios (Fabry et al. 2008).

A key question is how microbial communities and microbial-mediated biogeochemical processes will be affected by ocean acidification. Joint et al. (2011) recently argued that given that microbial assemblages have always experienced variable pH conditions, the appropriate null hypothesis to be tested is that “there will be no catastrophic changes in marine biogeochemical processes driven by phytoplankton, bacteria, and archaea.” In response to this article, Liu et al. (2010), performed a meta-analysis of published data and suggested that changes in microbial structure and function are possible. Both authors provide valid arguments to a complex issue that we have only just started to understand. So far, existing studies suggest that microbial-mediated processes such as carbon and nitrogen cycles may be affected. For example, Riebesell et al. (2007) showed that a phytoplankton community responded to higher CO_2_ concentrations (three times the present *p*CO_2_ conditions) in seawater an up to 39% increase in net primary production. Increased *p*CO_2_ may also impact the nitrogen cycle. The filamentous cyanobacterium *Trichodesmium*, a major contributor of new nitrogen in oligotrophic oceans, has been shown to increase carbon and nitrogen fixation rates by 35% to 100% at *p*CO_2_ levels predicted for 2100 (Hutchins et al. 2007). In addition to nitrogen fixation, other components of the nitrogen cycle may also be altered by ocean acidification. Nitrification can be affected by pH-driven changes in the availability of ammonia (NH_3_). Beman et al. (2010) suggest that a reduction in nitrification rates of 3–44% can occur within a few decades. With respect to bacterial communities there is little information and the existing studies are less clear. Most of the studies regarding bacteria under ocean acidification scenarios have been performed in large pelagic mesocosm systems that study the effect of carbonate chemistry modifications through the food web. These experiments are capable of realistic simulations where indirect effects from interactions with phytoplankton can be studied. Experiments such as these have demonstrated that bacterial abundance and activity can vary due to phytoplankton shifts under high _P_CO_2_ (Grossart et al. 2003; Allgaier et al. 2008). Regarding community structure, large mesocosms and small-scale approaches have revealed contrasting effects. In large pelagic mesocosms, dominant bacterial community shifts were not related to *p*CO_2_ (Roy et al. 2012), whereas in small microcosm systems, pH levels predicted for the year 2100 had a significant impact on bacterial structure (Krause et al. 2012).

It is clear that we still have much to learn about microbial dynamics under elevated *p*CO_2_ levels, particularly with respect to the underlying mechanisms that trigger some of the observed trends. Furthermore, the impact of ocean acidification on microbial function needs to be addressed with more focus on local or regional conditions, as the magnitude of carbonate changes will vary across regions. For example, anthropogenic stressors exacerbate ocean acidification through the development of hypoxic and anoxic zones due to increased eutrophication in coastal and estuarine areas. Low oxygen waters are more acidic than ocean waters. In a model saline estuary the development of hypoxia is enough to reduce pH levels by more than 0.5 units (Howarth et al. 2011).

### Effects of UVR in marine microbial communities

Researchers have studied the effects of UVR for some decades. An important impetus for studying UVR was the concern for the ozone layer, which had been adversely affected by chlorofluorocarbons. Following implementation of the Montreal protocol that placed restrictions on ozone-depleting substances, ozone levels in the atmosphere are no longer declining (McKenzie et al. 2011). However, recovery of the stratospheric ozone layer to 1980s levels is not likely to occur in the next decades (Weatherhead and Andersen 2006). In fact, the area of the Antarctica ozone hole reached a maximum in 2006 (NASA 2009) and in 2011, a record destruction of the ozone layer over the Arctic was reported (Manney et al. 2011). Therefore, changes in UV radiation levels in the future will depend on changes in various atmospheric factors, besides total ozone, including clouds, aerosols, as well as surface reflectivity (or albedo), in some locations. Other factors, including tropospheric gaseous pollutants and stratospheric temperature, may also play a role (WMO 2011). Due to the complexity of factors influencing changes in UV radiation levels reaching the Earth's surface, future trends in UV radiation levels are uncertain and contrasting predictions exist. For example, while some predictive models indicate that by the 2090s mean erythemal UV levels will drop by up to 12% worldwide compared with values recorded in 1980 (Bais et al. 2011), other models indicate that UVB levels will increase in the Northern Hemisphere in response to reductions in the amount of aerosols and clouds (Hegglin and Shepherd 2009; Watanabe et al. 2011).

It is well known that the amount of UVR that reaches the Earth's surface has important consequences for aquatic ecosystems. UVR is the most photochemically reactive waveband of incident solar radiation and can have genotoxic, cytotoxic, and ontogenetic effects on aquatic organisms (Bancroft et al. 2007). It is commonly divided into three wavelength ranges: UV-A (315–400 nm), UV-B (280–315 nm), and UV-C (<290 nm). DNA absorbs only weakly at longer UV wavelengths (Jones et al. 1987). Thus, the biological effects of UV-A are usually considered indirect, resulting from intracellular generation of reactive oxygen species (ROS), which cause oxidative damage to lipids, proteins, and DNA (Pattison and Davies 2006). UV-C wavelengths are generally not deemed to be environmentally relevant, given that they are almost completely screened out of the atmosphere by oxygen and ozone. UV-B is the highest energy wavelength of solar radiation that reaches the Earth's surface and the UV wavelength that is mostly affected by shifts in the ozone layer (Andersen and Sarma 2002). UV-B radiation can cause damage to nearly all biomolecules by direct absorption or indirectly as a result of enhanced formation of ROS (Vincent and Neale 2000).

Environmental effects of UVR radiation are generally attenuated by protective strategies displayed by living organisms, such as avoidance, photochemical quenching, and repair. The overall stress imposed by UVR exposure thus reflects a balance between damage, repair, and the energetic costs of protection, while it may also affect energy consumption and the biochemical composition of cellular material, resulting in lower survival and growth rates (Vincent and Neale 2000). UVR represents an important stressor for bacteria in aquatic ecosystems, as their simple haploid genomes provide little or no functional redundancy (Garcia-Pichel 1994). In general, exposure to UV-B reduces extracellular enzymatic activities (Herndl et al., 1993, Santos et al. 2012), oxygen consumption (Joux et al., 2009), and leucine and thymidine incorporation (Sommaruga et al., 1997, Santos et al. 2012). Different bacterial groups have also been shown to vary in their sensitivity to UVR and the potential to repair UVR-induced damage (Fernández Zenoff et al. 2006; Santos et al. 2012). *Gammaproteobacteria* have been identified as the most UV-resistant group in several aquatic environments (Alonso-Sáez et al. 2006; Ordoñez et al. 2009; Santos et al. 2012). Field studies have also identified the *Bacteroidetes* group as UV resistant (Alonso-Sáez et al. 2006; Fernández Zenoff et al. 2006). The *Alphaproteobacteria* group, on the other hand, has been reported to be UV-sensitive (Alonso-Sáez et al. 2006). Among *Alphaproteobacteria*, the SAR11 cluster, which is potentially the most abundant and ubiquitous clade of heterotrophic marine bacteria in the oceans (Morris et al. 2002) were found to be particularly sensitive to solar UVR (Alonso-Sáez et al. 2006; Ruiz-González et al. 2012). High UVR sensitivity of SAR11 was attributed to the high A+T content (69%) reported for the genome of the representative member of this group, *Pelagibacter ubique* (Giovannoni et al. 2005b). The UV sensitivity of the SAR11 group is also supported by recent observations of the disappearance of sequences affiliated to *Pelagibacter* in natural Patagonian bacterioplankton communities following an 8-day exposure to PAR, PAR+UVA, and PAR+UVA+UVB (Manrique et al. 2012). Other studies, however, indicate stimulation of SAR11 activity by light, potentially associated with the presence of proteorhodopsins (Giovannoni et al. 2005a; Mary et al. 2008; Lami et al. 2009). Further studies are necessary to elucidate how environmental factors, particularly UVR, affect SAR11 diversity and activity.

The differential sensitivity to UVR exhibited by the most abundant bacterial groups present in the bacterioplankton is of paramount importance for the biogeochemical impact of enhanced UVR on ecosystems. The rationale for this assumption is the contrasting activity displayed by different groups of bacteria on the utilization of DOM (Cottrell and Kirchman 2000). For example, UVR-sensitive *Alphaproteobacteria* populations seem to be responsible for a large part of low-molecular-weight DOM uptake, while the more UVR-resistant *Bacteroidetes* tend specialize in high-molecular weight DOM uptake (Cottrell and Kirchman 2000; Alonso-Sáez et al. 2006). Therefore, changes in bacterial community structure triggered by increased UV-B levels may promote dramatic shifts in DOM pathways (Morris et al. 2002).

## Synergistic Effect of UVR, Ocean Acidification, and Anthropogenic Pollutants

### Will interactions between UVR, ocean acidification, and anthropogenic pollutants affect marine microbes?

Despite the fact that several studies have shown that environmental, physical, and chemical parameters directly affect the toxicity of anthropogenic pollutants, the interactive effects of UVR and ocean acidification on the chemistry of these pollutants and their effects on marine microbial communities have received very little attention. Increased UVR levels and changes to ocean pH will certainly affect the chemistry of several natural compounds and environmental pollutants, thus altering the way that they will interact with marine organisms. Polycyclic aromatic hydrocarbons (PAH), one of the most common compounds associated to OH pollution, are ideal examples of photoactive contaminants that are strongly absorbed in the UV-A and UV-B spectral regions. It is known that PAH toxicity to marine organisms may increase with exposure to UVR. This increase is largely regulated by two processes: namely, photosensitization and photooxidation reactions. Both of these processes have the potential to release phototoxic aromatic hydrocarbons into the environment, which are more toxic than their parent compounds (Krylov et al. 1997).

Reduced oceanic pH has the potential to affect the adsorption of metals by organic particles. Generally, organic particles are negatively charged and, as pH declines, surface sites become less available to adsorb positive ions like metals (Millero et al. 2009). This aspect is particularly important due to the fact that more than 99% of the total concentration of most metals in seawater corresponds to organic complexes (Millero et al. 2009). Small deviations in the concentration of elements such as Cu and Cd can have a serious effect on the health of marine organisms (Millero et al. 2009). Organic materials though are often nonhomogeneous and of unknown structure. It is essential that we gain a better understanding of metal speciation in organic complexes (Doney et al. 2009; Millero et al. 2009).

A key question is whether there is any evidence of an interaction between UVR and ocean acidification on the one hand and anthropogenic pollutants on the other. The answer is yes, although several details are missing and require further research. Peachey (2005), for example, observed no significant effect of PAH and UVR exposure on larval crab mortality when exposed independently; the combined effect of both, however, resulted in up to 100% mortality. Photoenhanced toxicity of PAH due to UVR exposure has already been observed in a variety of organisms (Peachey 2005). This phenomenon has also been verified in isolated bacterial strains (McConkey et al. 1997), although only a few studies have addressed the effect of UVR photo-modified pollutants in complex microbial assemblages (Pelletier et al. 2006; Petersen et al. 2008). In a microcosm experiment designed to study the effects of increased UV-B in the presence of the water soluble fraction of crude oil, an increase in mortality was observed in the phytoplankton community exposed to UV-B. In this scenario, the toxic effects on phytoplankton led to a release of carbon and other nutrients that stimulated bacterial growth (Pelletier et al. 2006). Petersen et al. (2008) reported a similar effect in sediment stocked in microcosms. Algal ^14^C-incorporation and chlorophyll *a* content both declined in sediments exposed to UV-light and pyrene. At the same time, oxygen consumption and the release of N and P increased, suggesting an increase in bacterial activity (Petersen et al. 2008).

Fabry et al. (2008) suggested that altered water CO_2_ chemistry in combination with other environmental stressors may modify the responses of organisms, and even ecosystems, to these stressors in ways that differ substantially from the action of only a single stressor. Indeed, ocean acidification in combination with elevated nutrient inputs can accelerate the expansion of filamentous turfs at the expense of calcifying algae in a synergistic response 34% greater than the sum of their individual effects (Russell et al. 2009). Recently, Roberts et al. (2013) study showed that amphipod DNA damage was 2.7 times higher in metal-contaminated sediment under an increased *p*CO_2_ (750 μatm) scenario (Roberts et al. 2013). However, nothing is known about possible changes in the response of marine microbes to anthropogenic pollutants under increased *p*CO_2_ scenarios.

Very little is known about the potential interactions (antagonistic, additive, or synergistic) between different pollutants. Although it lies outside the scope of this review, this topic is important given that marine ecosystems are exposed to a myriad of novel chemical substances that can react in unexpected ways (Crain et al. 2008).

### Microbial-mediated detoxification

An important question that we need to address is whether the effects of ocean acidification and UVR will affect microbial communities in such a way that it may alter microbial-mediated detoxification of anthropogenic pollutants. In addition to directly measurable physiological effects on marine microbes, UVR and ocean acidification can also indirectly affect the toxicity of anthropogenic pollutants by inducing shifts in microbial community structure; they can also alter microbial-mediated detoxification processes. For example, the bioavailability of inorganic nutrients required for bacterial growth, such as nitrogen and phosphorus, is a key factor in successful ecosystem detoxification of PAH (Atlas and Bartha 1972). Anything that alters the nitrogen cycle, such as ocean acidification has the potential to alter microbial-mediated PAH detoxification processes and consequently PAH toxicity.

**Figure 2:**
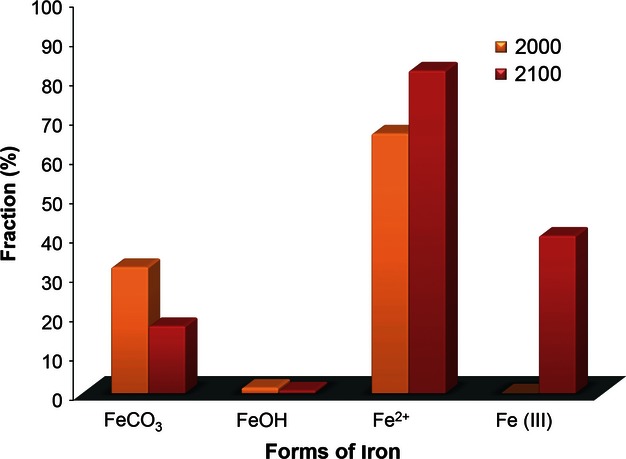
One hundred years scenario of forms of iron in surface ocean waters considering the 0.4 pH units decrease modeled by Caldeira and Wickett (2003), at 25°C and salinity of 35. Adapted from Millero et al. (2009). As coastal and estuarine areas are very different between them, modeling on speciation of metals in these areas is needed for a more complete and accurate scenarios.

A critical potential effect of ocean acidification is an alteration of metal bioavailability. Metals interact with microbes in various ways, and are involved in virtually all aspects of microbial growth and metabolism (Gadd 2010). Changes in iron chemistry are particularly important, given that iron is a limiting nutrient for marine phytoplankton in large oceanic regions (Sunda 2010). Shi et al. (2010) reported that the predicted *p*CO_2_ level for the year 2100 would reduce iron uptake by diatoms and coccolithophores by 10–20%. The reduction in iron availability is believed to be related to pH-induced binding of iron to organic ligands, thus reducing biologically available Fe(III) (Shi et al. 2010; Sunda 2010). The net effect of ocean acidification on iron chemistry is still unclear. For example, although lower pH also increases iron binding to organic ligands, the solubility of Fe(III) increases with water acidification in surface ocean waters (Fig. 2) (Millero et al. 2009).

Shi et al. (2010) highlight the potentially harmful effects of iron bioavailability due to ocean acidification. Iron is also an important factor in the detoxification of hydrocarbons, by influencing the activity of enzymes that catalyze the oxidative breakdown of PAH (Dinkla et al. 2001; Santos et al. 2008). Monooxygenase and dioxygenase enzymes, essential in most microbial PAH degradation pathways, require a metal cofactor which is often iron (Bugg 2003). The activity of several key enzymes, including toluene monooxygenase, in the degradation of the aromatic hydrocarbon toluene by *Pseudomonas putida*, was found to be reduced under iron-limiting conditions (Dinkla et al. 2001). The complexity of biological, chemical, and environmental interactions in natural environments restricts our ability to establish cause–effect relationships. Understanding the interactive effects of climate change and anthropogenic pollutants on microbial communities is a complex task. It requires the study of a multitude of chemical and biological pathways, which may only be experimentally addressed in detail under controlled conditions.

## Microcosm Coupled With Molecular Biology Technologies as an Experimental Framework

It is important that we obtain a mechanistic understanding of the effects of ocean acidification and UVR on pollutant toxicity and degradation. Pollution events, however, occur over a wide range of spatial and temporal scales making it difficult to gauge cause–effect relationships. This difficulty is enhanced when studying additional levels of complexity such as interactive effects with different stressors. In such cases, small-scale models, such as microcosms and mesocosms, can be useful tools.

Microcosms and mesocosms are simplified systems, constructed to mimic natural environments under controlled conditions (Roeselers et al. 2006). Both simplified systems are powerful tools that have facilitated the study of several ecological processes, including research on predator–prey coevolution, ecosystem level selection, resource competition, and adaptive radiation (Jessup et al. 2004). Both systems have advantages and disadvantages (Table 1). The major advantage of these setups is that they enable a high degree of experimental control and replication. The level of control provided is virtually impossible to obtain through standard field surveys (Benton et al. 2007). In addition to this, microcosms and mesocosms enable researchers to experiment with highly toxic substances that would not be possible in situ. While there is some concern that these models are too small, both spatially and temporally, to be useful, the goal of these experiments is not to fully reproduce nature in a laboratory model system, but rather to simplify complex ecosystems so that essential dynamics can be captured (Jessup et al. 2004). The distinction between microcosms and mesocosms is somewhat arbitrary. Mesocosms tend to be outdoor and larger in size, increasing biological and spatial complexity (Petchey et al. 2002), but diminishing experimental control and reducing replicability. In contrast, small microcosm setups allow a degree of experimental control and replication that is difficult to achieve with larger outdoor mesocosms.

**Table 1:** Main advantages and disadvantages of microcosm and mesocosm experiments

	Environment	Microcosm	Mesocosm
Ease of replication	+	+++	++
Precise control over environmental parameters	+	+++	++
Treatments under investigation can be highly controlled	+	+++	++
Space and temporal scale	+++	+	++
Multitrophic interactions	+++	++	+++
Functional ecosystem mimicry	+++	++	++
Circumvent oversimplification	+++	+	++

+, limited; ++, moderate; +++, full control.

Of course, the size and design of these experiments will always depend on the research question. Research on potential interactions between climate change and anthropogenic pollutants can greatly benefit from the experimental control of small microcosm systems. Many field studies only provide correlative evidence of certain phenomena. Microcosm experiments can help to elucidate whether there is an actual mechanistic effect. Additionally, small-scale experiments with microbes can overcome microcosm scale-related limitations associated with studying larger organisms. Due to the small size and short generation times of microbes, it is possible to simulate complex temporal and spatial scales within microcosms (Jessup et al. 2004). Climate change interactions with anthropogenic pollutants could be tested over several generations. For example, Collins and Bell (2006) simulated evolutionary responses of *Chlamydomonas* populations exposed to increasing concentrations of CO_2_ using microcosm experiments. Nevertheless, temporal and spatial scales should be considered with care when performing experiments with microbes. Enclosure within small experimental containers can induce shifts in microbial communities known as the “bottle effect” (Ferguson et al. 1984). It is important to monitor microbial communities and determine the extent to which results are biased by microcosm enclosure.

Currently, one of the key gaps in our understanding of how climate change may affect microbial communities is the lack of microcosm systems designed to simulate predicted climate change scenarios in marine environments. For example, the effect of carbonate chemistry manipulation on microbial communities has mainly been assessed in larger mesocosms. Reliable microcosm systems designed to mimic fundamental dynamics of marine environments and capable of simulating climate change scenarios are needed. However, developing microcosm systems capable of simulating climate change scenarios such as increased UVR or ocean acidification is not a trivial task. For example, the spectral irradiance emitted by UVR lamps does not match natural solar irradiance. UVR lamps emit more short-wave and less long-wave UVR than the sun (Xu and Sullivan 2010). A possible solution is to calibrate the lamps with a biological spectral weighting function that describes the effectiveness of lamp wavelength to produce biological responses (Andreasson and Wängberg 2006). However, this experimental setup is complex for long-term outdoor microcosm experiments as UVR lamps must be continuously calibrated in order to account for daily and seasonal light variation. If the experimental setup does not involve wavelength isolation, microcosms can be directly exposed to sunlight. Recently, Gao et al. (2012) exposed microcosms directly to several levels of solar radiation to simulate the synergistic effects of light exposure and increased *p*CO_2_ in phytoplankton at different depths. Likewise, there are several methods that simulate future changes in seawater chemistry. CO_2_ bubbling, addition of high-CO_2_ seawater, and combined addition of acid and HCO_3_^-^ are the three approaches that most closely mimic future scenarios of shifts in seawater chemistry (Gattuso and Lavigne 2009). Probably, the easiest to implement in microcosms is CO_2_ bubbling with pH stats systems. In these systems, pH is monitored continuously and a controller valve increases or reduces the addition of CO_2_ when pH deviates from a set value (Gattuso and Lavigne 2009).

**Figure 3:**
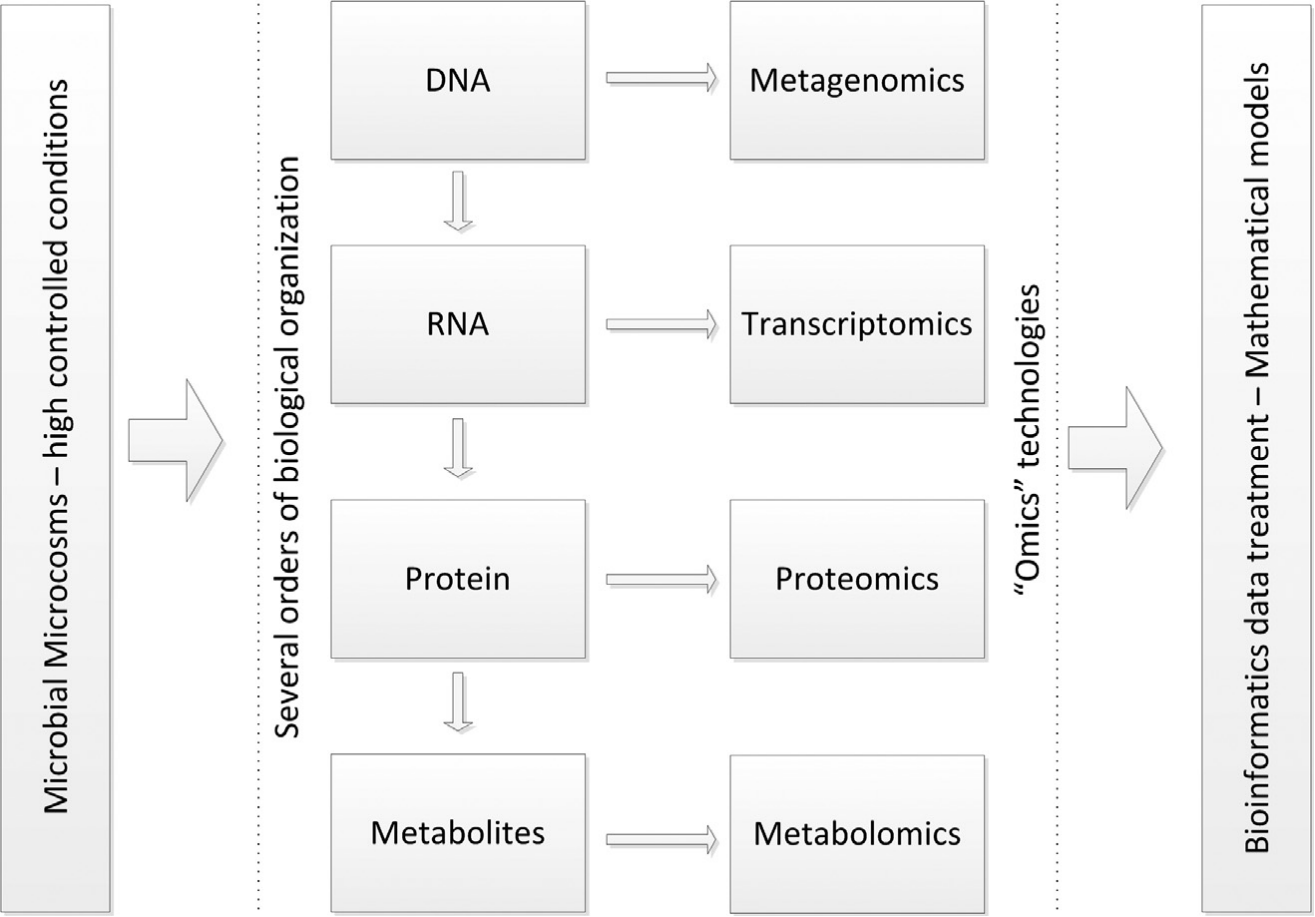
Microbial microcosm coupled with “omics” technologies can provide an excellent tool to gain mechanistic insights into climate change and anthropogenic pollution interactive effects at several levels of biological organization.

As referred to above, the use of microcosms to address fundamental ecological questions is not new. However, this approach combined with recent advances in microbe characterization technologies can provide an important framework to start unraveling how climate change and pollution may interact to affect several levels of biological organization (Fig. 3). The recent development of molecular technologies has enabled scientists to assess the structure and function of microbes in a range of different environments including soil, sediment, water, and within animal and plant hosts. The development of high-throughput DNA sequencing technologies has been a milestone in the field of metagenomics and metatranscriptomics. Metagenomic analysis, however, only provides structural and putative functional information of the microbes under study. In order to restrict focus to the active (as opposed to dormant, Urich et al. 2008) members of microbial communities and the genes expressed, several protocols were developed to sequence actively transcribed RNA and messenger RNA (mRNA) (also known as metatranscriptomics). Metatranscriptomic analysis can facilitate the study of microbial responses to rapid environmental change (e.g., an oil spill), thereby linking structural shifts to community function (Mason et al. 2012). In parallel, advances in efficient chromatographic separation coupled with mass spectrophotometry-based approaches have enabled high-throughput protein identification. The study of the entire set of proteins (proteome) produced by a given microbial community in a particular environment has led to a new field known as metaproteomics. Together with metagenomics and metatranscriptomics, metaproteomics can facilitate the study of cellular responses to changing environmental conditions. Metabolite profiling in complex biological samples is also an emerging field. This new approach, known as metabolomics, involves the quantitative and qualitative analysis of the complete set of metabolites present in a sample, providing additional information on metabolic and physiological potential.

While these technologies have greatly improved our ability to acquire data, they have also created new challenges. The current rapid development of “omic” technologies is unique in that it actually exceeds the rate of chip performance evolution in the computing industry, also known as Moore's law (Gilbert et al. 2012). However, our ability to integrate the large amount of “omic” data is maturing rapidly. For example, the construction of co-occurence and correlation networks from presence–absence or abundance data, in a process known as network inference, is being increasingly used to predict microbial interactions (Faust and Raes 2012). Recently, Larsen et al. (2012) used an artificial neural network to develop a model that predicts the abundance of microbial taxa as a function of environmental conditions and biological interactions. This method can be seen as a first step in the application of bioclimatic modeling to predict microbial community and environmental interactions under future global change scenarios.

Microcosm simulations must be developed in line with data acquisition from the field and modeling. Microbial observatories that generate long-term data series from different habitats and across several gradients (e.g., polluted vs. nonpolluted areas or areas of volcanic activity where CO_2_ gas is released into the water creating a natural gradient of pH levels; Hall-Spencer et al. 2008) can also provide valuable information on potential interactive effects. Furthermore, data series such as these can be used to confirm or refute hypotheses formulated from microcosm experiments.

## Concluding Remarks

Understanding the full extent of interactive effects of global climate change and pollutants on microbes is a complex task, which entails the study of a multitude of interactions. In addition to this, the information about these interactions is scarce and studies in this field are still in their infancy. Further studies are needed to evaluate how the effects of oceanic pH and UV radiation (UVR) will affect microbial detoxification processes in marine ecosystems. The experimental data gathered so far allow us to predict that independent and interactive effects of UVR and ocean acidification will probably affect microbial community structure and function. Given the importance of microbial-mediated processes, there is a potential for the disruption of key ecosystem services. At present, there are some major technical challenges that still need to be met with respect to reliable and replicable integrated approaches to simulate predicted climate change scenarios and evaluate how they will affect the toxicity of pollutants and the functioning of microbial communities. This endeavor demands statistically robust experiments under controlled conditions, where biological and nonbiological markers of environmental function can be accurately identified and quantified. Microcosm experiments paired with new “omics” technologies, along with field surveys can provide an excellent framework to ascertain the effect of anthropogenic pollutant toxicity and microbial function under different climate change scenarios. It is important that we start to identify interactions resulting from global climate change and anthropogenic pollution in order to mitigate known and novel environmental threats.
